# Out-of-Frame Mutations in *ACTN2* Last Exon Cause a Dominant Distal Myopathy With Facial Weakness

**DOI:** 10.1212/NXG.0000000000000619

**Published:** 2021-08-10

**Authors:** Marco Savarese, Anna Vihola, Manu E. Jokela, Sanna Pauliina Huovinen, Simonetta Gerevini, Annalaura Torella, Mridul Johari, Marina Scarlato, Per Harald Jonson, Maria Elena Onore, Peter Hackman, Mathias Gautel, Vincenzo Nigro, Stefano Carlo Previtali, Bjarne Udd

**Affiliations:** From the Folkhälsan Research Center (M. Savarese, A.V., M.J., P.H.J., P.H., B.U.), Helsinki; Department of Medical Genetics (M. Savarese, A.V., M.J., P.H.J., P.H., B.U.), Medicum, University of Helsinki; Neuromuscular Research Center (A.V.), Department of Genetics, Fimlab Laboratories, Tampere; Division of Clinical Neurosciences (M.E.J.), Department of Neurology, Turku University and University Hospital; Neuromuscular Research Center (S.P.H.), Department of Pathology, Fimlab Laboratories, Tampere, Finland; Neuroradiology Unit (S.G.), ASST Papa Giovanni XXIII, Bergamo; Dipartimento di Medicina di Precisione (A.T., M.E.O., V.N.), Università degli Studi della Campania “Luigi Vanvitelli,” Napoli; Telethon Institute of Genetics and Medicine (A.T., V.N.), Pozzuoli; Division of Neuroscience and U.O. Neurologia (M. Scarlato, S.C.P.), IRCCS Ospedale San Raffaele, Milano, Italy; Randall Centre for Cell and Molecular Biophysics (M.G.), King's College London BHF Centre of Research Excellence, United Kingdom; Department of Neurology (B.U.), Vaasa Central Hospital; and Neuromuscular Research Center (M.E.J., B.U.), Department of Neurology, Tampere University and University Hospital, Finland.

## Abstract

**Background and Objectives:**

To clinically, genetically, and histopathologically characterize patients presenting with an unusual combination of distal myopathy and facial weakness, without involvement of upper limb or shoulder girdle muscles.

**Methods:**

Two families with a novel form of actininopathy were identified. Patients had been followed up over 10 years. Their molecular genetic diagnosis was not clear after extensive investigations, including analysis of candidate genes and FSHD1-related D4Z4 repeats.

**Results:**

Patients shared a similar clinical phenotype and a common pattern of muscle involvement. They presented with a very slowly progressive myopathy involving anterior lower leg and facial muscles. Muscle MRI finding showed complete fat replacement of anterolateral compartment muscles of the lower legs with variable involvement of soleus and gastrocnemius but sparing thigh muscles. Muscle biopsy showed internalized nuclei, myofibrillar disorganization, and rimmed vacuoles. High-throughput sequencing identified in each proband a heterozygous single nucleotide deletion (c.2558del and c.2567del) in the last exon of the *ACTN2* gene. The deletions are predicted to lead to a novel but unstructured slightly extended C-terminal amino acid sequence.

**Discussion:**

Our findings indicate an unusual form of actininopathy with specific molecular and clinical features. Actininopathy should be considered in the differential diagnosis of distal myopathy combined with facial weakness.

Distal myopathies are genetic muscle diseases, presenting at the onset with weakness of foot and lower leg and/or hand and forearm muscles, which cause progressive loss of muscle tissue.^1^ Prominent anterior lower leg weakness, facial weakness, and scapular winging are the hallmarks of facioscapulohumeral muscular dystrophy.^2^ Facial weakness has also been reported in patients with distal myopathies due to mutations in *ADSSL1*, *RYR1*, *MYH7*, *NEB*, and *DNM2*.^1^

We have been following up for many years 2 unrelated patients with an unusual combination of distal lower limb myopathy and facial weakness without involvement of upper limbs or shoulder girdles. Proband 1 (F1,II:4) is a Finnish woman in her 60s (eFigure1, links.lww.com/NXG/A442). The proband's first neurologic examination in 2005 revealed weakness of ankle dorsiflexion and mild atrophy of anterior lower leg muscles. Mild facial weakness, noticeable since her late childhood, was also observed (Table). Weakness slowly progressed over the years. At age 59 years, she displayed severe bilateral foot drop and a moderate, slightly asymmetric facial weakness of *frontalis*, *orbicularis oculi*, and perioral muscles without ptosis or external ophthalmoplegia. She did not show scapular winging or any proximal weakness. Echocardiography did not show any cardiac abnormalities. Proband's daughter (F1,III:2), currently in her 30s, had had conservatively treated scoliosis as a teenager. At around age 35 years, she developed mild bilateral foot drop and lower facial weakness with inability to whistle.

**Table:**
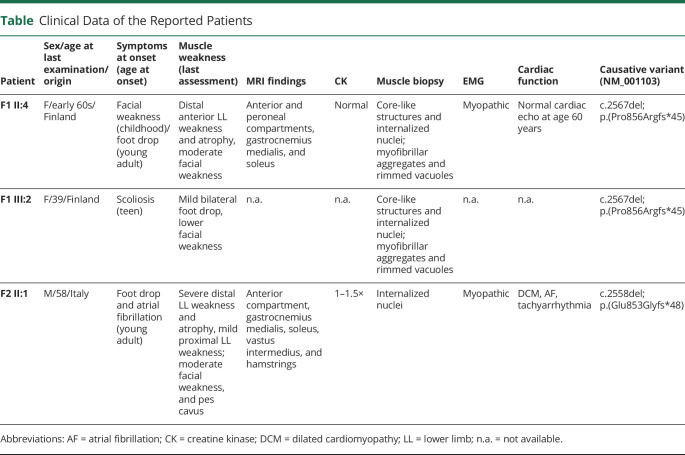
Clinical Data of the Reported Patients

The second proband (F2,II:2) is a 58-year-old Italian man (eFigure1). He had presented with tachyarrhythmia and lower limb distal weakness since his early adulthood (Table). The disease later progressed to proximal lower limb and facial muscles (eFigure2, links.lww.com/NXG/A443) without scapular and upper limbs involvement. He also developed dilated cardiomyopathy.

Lower leg muscle MRI finding showed a similar pattern with complete fatty replacement of anterolateral compartment muscles of the lower legs but largely sparing thigh muscles (Figure 1).

**Figure 1:**
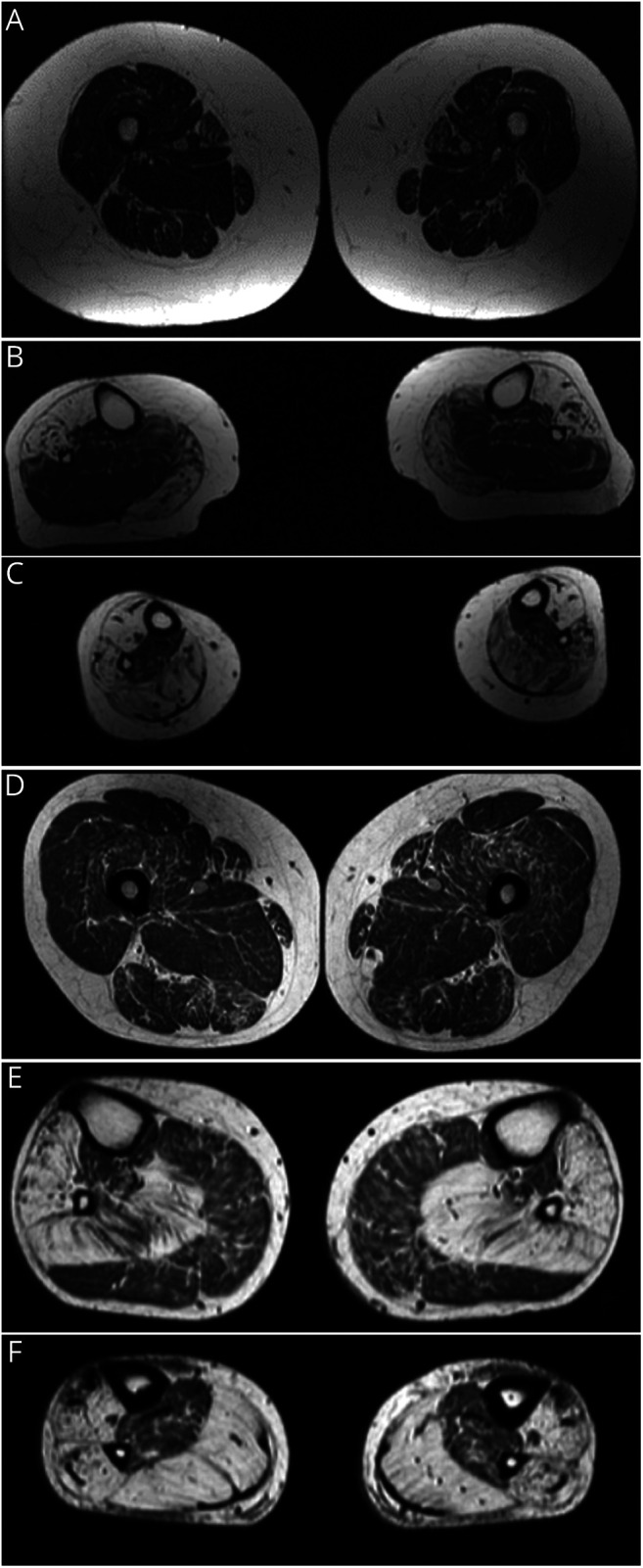
Muscle MRI Findings in the Finnish (A–C) and Italian (D–F) Probands (A) Thigh: normal; (B and C) lower leg: anterolateral compartment muscles, gastrocnemius medialis, and distal soleus muscles bilaterally are replaced by fibrofatty tissue. (D) Thigh: mild diffuse fatty degenerative changes in vastus intermedius and hamstring muscles; (E and F) lower leg: anterolateral compartment muscles and bilateral soleus muscles are completely replaced by fibrofatty tissue. Mild changes in gastrocnemius medialis.

The probands did not have any FSHD-1–causing mutation. High-throughput sequencing analysis (for F1,II:4, Nimblegen SeqcapEz Human Exome Library v2.0; Roche, Basel, Switzerland, and for F2,II:1, ClearSeq Inherited DiseaseXT; Agilent Technologies, Santa Clara, CA) identified single nucleotide deletions in the *ACTN2* last exon and did not detect causative mutations in *SMCHD1* or other myopathy-causing genes. The variant NM001103:c.2567del in the Finnish patients causes a frameshift predicted to replace the last 42 amino acids with 44 novel (p.Pro856Argfs*45) (eFigure3, links.lww.com/NXG/A444). The variant segregated with the disease in the proband's daughter and was absent in the unaffected relatives tested. The *ACTN2* deletion, NM001103:c.2558del, identified in the Italian patient, replaces the 45 final amino acids (p.Glu853Glyfs*48) and results in a C-terminal amino acid sequence similar to the one resulting from the c.2567del variant (eFigure3). The variant was not present in the proband's healthy mother and brother.

The identified variants are not listed in gnomADv2.1.1 and are not anticipated to result in nonsense-mediated decay. The variants replace the entire second EF (EF3-4) domain that is needed for alpha-actinin 2 dimerization and for its binding to titin^3^ (eFigure3).

Muscle biopsies showed internalized nuclei and fiber size variation. Immunohistochemical analysis was performed on the Finnish patients using monoclonal antibodies against desmin (Biogenex, Fremont, CA; clone D33), myotilin (Leica Biosystems, Wetzlar, Germany; clone RSO34), alpha-B-crystallin (Novus Biologicals, Littleton, CO; clone 1D11C6E6), and alpha-actinin (Sigma-Aldrich, St. Louis, MO; clone EA-53). In the Finnish patients' biopsies, there were rimmed vacuoles and myofibrillar aggregates, strongly positive for alpha-crystallin and myotilin and weakly positive for desmin (Figure 2). Nicotinamide adenine dinucleotide stain showed core-like pathology.

**Figure 2:**
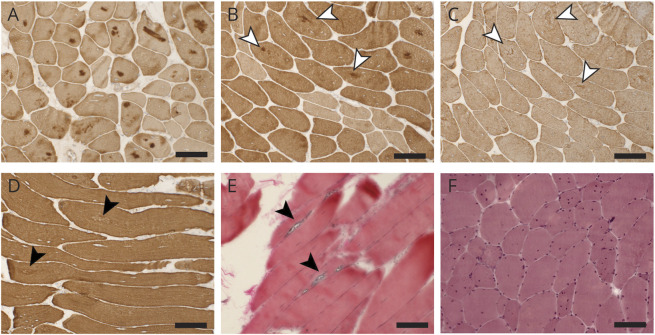
Histopathology Immunohistochemical stainings of the muscle biopsy of the Finnish proband (A–D) show central myofibrillar aggregations in several fibers positive for alpha-B-crystallin (A) and myotilin (B), whereas desmin (C) shows weak positivity; B and C are serial sections, and the same fibers are indicated with white arrowheads. Alpha-actinin staining (D) shows areas of myofibrillar disorganization, indicated by arrowheads. Herovici staining (E) of the muscle biopsy of the daughter of the Finnish proband shows 2 fibers with prominent rimmed vacuoles (arrowheads). Hematoxylin and eosin staining of the muscle biopsy of the Italian proband (F) shows fiber size variation and multiple internal nuclei. Scale bar = 100 μm.

In the Finnish proband's muscle biopsy, immunochemistry showed minor irregular staining of alpha-actinin pinpointing the areas of myofibrillar disarray (Figure 2), which, however, could simply reflect disorganization of the underlying myofibrils. No clear accumulation of alpha-actinin was observed (the antibody recognizes both alpha-actinin 2 and 3, but the patient has no expression of alpha-actinin 3, being homozygous for the *ACTN3* p.R577X variant^4^). A transcriptome analysis (library prepared using the NEBNext Ultra II Directional RNA library Prep for Illumina, New England Biolabs) on the same sample confirms that the variant c.2567del results in a normal, biallelic expression of *ACTN2* transcripts.

In 2019, we described a distal myopathy, without facial weakness, caused by *ACTN2* missense variants in 4 families.^5^ De novo *ACTN2* variants were identified in 2 patients with congenital myopathy with structured cores, showing mild facial weakness.^6^ Missense variants have also been associated with cardiomyopathies^7^ (eFigure3c, links.lww.com/NXG/A444).

In this study, we describe a novel form of dominant distal actininopathy to be considered in the differential diagnosis of patients having lower leg predominant distal myopathy with facial weakness.

## Standard Protocol Approvals and Patient Consents

Patients provided informed consent. Ethical approval falls under HUS:195/13/03/00/11.

## Data Availability

Deidentified data are available on request.
